# Prevalence and Utilization Patterns of Digital Medical Resources in Clinical Practice Among Family Medicine Physicians in Riyadh Health Clusters

**DOI:** 10.7759/cureus.108512

**Published:** 2026-05-08

**Authors:** Yasser S Aljaied, Saleh S Alsuwayt, Wid A Nawab, Hamoud M Almubki

**Affiliations:** 1 Family Medicine, King Saud Medical City, Riyadh, SAU; 2 Family Medicine, Dr. Sulaiman Al Habib Medical Group, Riyadh, SAU

**Keywords:** clinical decision-making, clinical knowledge support systems, digital medical resources, evidence-based clinical practice, family medicine, point-of-care tools, riyadh health clusters, saudi arabia

## Abstract

Background

The integration of digital medical resources has become increasingly important in supporting evidence-based clinical decision-making. In the context of Saudi Arabia’s healthcare transformation, understanding how family physicians utilize these resources during routine clinical practice remains limited.

Objective

To assess the prevalence, usage patterns, motivations, and perceived barriers related to the use of digital clinical information and decision-support resources during clinic hours among board-certified family medicine physicians working in Riyadh Health Clusters 1, 2, and 3.

Methods

A cross-sectional study was conducted between January and May 2025 among board-certified family medicine physicians in Riyadh Health Clusters 1, 2, and 3. Data were collected via a self-administered electronic survey distributed during structured site visits. Descriptive statistics and chi-square analyses were performed to examine associations between digital resource utilization and participant demographics.

Results

A total of 96 participants were included. The majority were aged 25-34 years (n = 61, 63.5%), and most were senior registrars (n = 52, 54.2%). Daily digital resource use was reported by 43 participants (44.8%), with UpToDate being the most utilized resource (n = 36, 37.5%), followed by AMBOSS (n = 19, 19.8%). Access to updated clinical information (n = 55, 57.3%) and improved decision-making (n = 37, 38.5%) were the primary motivations for usage. Younger age, fewer years of clinical experience, and job position were significantly associated with a higher frequency of digital resource utilization (p < 0.05). While most participants perceived positive impacts on clinical efficiency and patient outcomes, barriers such as workflow disruptions, time constraints, and financial limitations were identified.

Conclusion

Digital medical resources are widely integrated into clinical practice among family medicine physicians in the Riyadh Health Clusters. Utilization patterns vary by physician demographics, with younger and less experienced physicians demonstrating greater reliance. Institutional support, accessibility, and continuous professional development are essential for optimizing digital resource integration into family medicine practice.

## Introduction

The increasing accessibility and rapid expansion of digital medical resources have significantly transformed modern healthcare, particularly in the realm of clinical decision-making and evidence-based practice [1]. These point-of-care (POC) tools enable healthcare providers to access real-time, peer-reviewed clinical information, contributing to improved diagnostic accuracy, enhanced patient safety, and better clinical outcomes [2,3]. International evidence-based platforms such as UpToDate, DynaMed, BMJ Best Practice, AMBOSS, and MDCalc, alongside Saudi national initiatives, including the Family Medicine Guide Handbook, its associated website and eBook developed by the Saudi Society of Family and Community Medicine, as well as the recently launched Daleel FM platform, collectively support clinical practice among family physicians in Saudi Arabia by providing high-quality, evidence-based clinical guidance [4]. These platforms are now extensively utilized in routine clinical practice, offering immediate access to the latest clinical guidelines, comprehensive drug information, decision-support calculators, and up-to-date medical literature [5,6].

Family physicians, who serve as the first point of contact for a wide variety of health conditions, particularly benefit from these tools, given the comprehensive and diverse nature of family medicine practice [7]. The effective use of digital clinical knowledge systems has been associated with improved healthcare quality indicators. For example, Longhurst et al. demonstrated that hospitals utilizing UpToDate had significantly shorter patient lengths of stay and lower inpatient mortality rates compared with non-users. Similarly, Wager et al. reported reductions in patient complications with the adoption of clinical knowledge support systems [2]. Beyond improving patient outcomes, digital resources also enhance efficiency at the point of care; Schilling et al. found that clinicians were able to answer 84% of their clinical questions using UpToDate within an average of five minutes, outperforming traditional literature searches such as PubMed [3].

While global studies show widespread adoption of digital resources among healthcare professionals, the extent of usage varies by region and healthcare system maturity. In Saudi Arabia, healthcare digitalization has accelerated, particularly following the Vision 2030 initiatives, leading to rapid telehealth expansion and the widespread integration of digital health platforms [8]. However, despite national investment in healthcare infrastructure, studies have revealed variable awareness and adoption rates of specific digital resources among Saudi healthcare providers. Ahmed et al. found that among Ministry of Health staff in Saudi Arabia, only 39% were aware of UpToDate and 19% of BMJ Best Practice, despite these being widely accessible internationally [6].

Locally, Alturki et al. reported that while 93% of family physicians in Riyadh used internet sources for clinical questions, less than half reported accessing formal evidence-based medicine (EBM) databases [7]. Several factors may influence this utilization pattern, including time constraints, limited training, poor system usability, and technological barriers, as identified by Aljarboa and Miah in a recent qualitative study of Saudi primary care physicians [9].

These barriers highlight the importance of investigating not only the prevalence but also the determinants of digital resource usage in clinical practice.

Digital medical resources have become integral to evidence-based practice, providing real-time clinical information and decision support at the point of care. In Saudi Arabia’s evolving healthcare system, such tools hold particular importance for enhancing quality, efficiency, and patient outcomes. Family physicians, as primary care providers, are especially reliant on these resources during clinic hours. However, data on their utilization among family physicians in Saudi Arabia remain limited. Therefore, this study aimed to provide a cross-sectional snapshot of the adoption and utilization of digital clinical information and decision-support resources, including platforms such as UpToDate, during clinic hours among board-certified family medicine physicians working in secondary and tertiary healthcare facilities within Riyadh Health Clusters 1, 2, and 3 between January and May 2025.

Objectives

The objective of this study was to assess the prevalence, usage patterns, motivations, and barriers associated with the use of digital medical resources during clinic hours among family medicine physicians practicing in the Riyadh Health Clusters. Specifically, the study examined the overall prevalence of digital resource utilization during clinical encounters, identified the most commonly used types of digital resources, and explored the underlying reasons and motivations for their use. Additionally, the study assessed physicians’ perceptions regarding the impact of digital resource utilization on healthcare delivery efficiency and effectiveness, as well as their overall attitudes toward integrating these resources into clinical practice. Potential barriers and challenges associated with adopting digital resources were also investigated. Finally, the study evaluated physicians’ level of confidence in carrying out clinical duties without access to digital medical resources.

## Materials and methods

Study design and setting

A cross-sectional study was conducted between January and May 2025 to provide a snapshot of the adoption and utilization of digital clinical information and decision-support resources during routine clinic hours. The study included board-certified family medicine physicians employed at secondary and tertiary healthcare facilities within Riyadh Health Clusters 1, 2, and 3, located in Riyadh, Saudi Arabia.

Study population

Eligible participants included all board-certified family medicine physicians actively practicing within the designated Riyadh Health Clusters. Exclusion criteria included non-board-certified physicians, including family medicine residents, general practitioners, and individuals who had completed family medicine residency training but had not successfully passed the final Objective Structured Clinical Examination (OSCE).

Sample size and sampling procedure

The total target population consisted of 127 board-certified family medicine physicians across the three Riyadh Health Clusters. The minimum required sample size was calculated using a 95% confidence level and a 5% margin of error, yielding a target sample size of 96 participants to ensure sufficient statistical power and representativeness.

Although random probability sampling was not employed, a structured time-based sampling approach was utilized. Over the five-month data collection period, systematic site visits were conducted across various healthcare facilities within the Riyadh Health Clusters. Visits were scheduled on different weekdays and shifts to capture physicians working across varying clinical schedules and settings. Eligible physicians who were present at the time of the visit were invited to participate.

Data collection

During clinic visits, participants were invited to complete a self-administered electronic questionnaire via a secure Google Forms link. The questionnaire was developed specifically for this study in collaboration with experts in family medicine and health informatics.

Prior to deployment, the instrument was pilot tested among 10 board-certified family medicine physicians who were not included in the final study sample. Feedback from this pilot phase informed minor revisions to enhance the clarity, content validity, and overall comprehensiveness of the tool.

The finalized version of the questionnaire is available in Appendix 1.

The data collectors remained on-site to address any questions, verify eligibility, and encourage real-time completion of the survey to minimize non-response bias. This approach allowed for immediate data capture while preserving the confidentiality and autonomy of participants.

Statistical analysis

Data entry and statistical analyses were performed using IBM SPSS Statistics, version 29.0.2.0 (IBM Corp., Armonk, NY, USA). Categorical variables were summarized using frequencies and percentages. The primary descriptive outcomes included frequency of digital resource use, type of digital resource used, motivations for use, perceived benefits, attitudes toward integration, confidence without digital access, and perceived barriers.

Univariate associations between categorical variables were assessed using the chi-square test. The main independent variables examined were age group, gender, job position, and years of clinical experience. The main dependent variables were digital resource usage frequency, type of resource used, duration of use, reasons for use, perceived impact, confidence without access, and reported barriers. Prevalence estimates of the primary outcomes were reported with corresponding frequencies, percentages, and 95% confidence intervals, where applicable. A two-tailed p-value of <0.05 was considered statistically significant. No imputation was performed, as only completed survey responses were included in the final analysis.

## Results

A total of 96 participants were included in this study. The demographic and professional characteristics of the participants are presented in Table 1. Most participants were aged 25-34 years, 61 (63.5%), and males represented the majority of the sample, 55 (57.3%). Based on the Saudi Commission for Health Specialties classification, Senior Registrars represented the largest professional group, 52 (54.2%). Most participants had 1-5 years of clinical experience, 50 (52.0%).

**Table 1:** Participants’ demographic and professional characteristics (N = 96) *SCFHS: Saudi Commission for Health Specialties

Characteristic	Category	n (%)
Age distribution of participants	25-34 years	61 (63.5%)
	35-45 years	26 (27.1%)
	46-55 years	7 (7.3%)
	>56 years	2 (2.1%)
Gender of participants	Male	55 (57.3%)
	Female	41 (42.7%)
Job position (SCFHS* classification)	Senior registrar	52 (54.2%)
	Consultant	44 (45.8%)
Clinical experience distribution of participants	<1 year	12 (12.5%)
	1-5 years	50 (52.0%)
	6-15 years	23 (24.0%)
	16-25 years	9 (9.4%)
	>25 years	2 (2.1%)

The descriptive characteristics and digital medical resource utilization patterns are summarized in Table 2. Daily use during clinics was the predominant pattern, 43 (44.8%). The most frequently reported reason for selecting specific digital medical resources was the availability of illustrative materials, including algorithms and flowcharts, 36 (37.5%). The most common duration of use per session was 4-6 minutes, 55 (57.3%). UpToDate was the most commonly reported digital medical resource, 36 (37.5%). The primary reason for using digital medical resources was access to current medical information, 55 (57.3%), while quality improvement and patient safety were the most frequently reported motivators, 48 (50.0%). Agreement that Continuing Medical Education credits influenced digital medical resource use was reported by 51 participants (53.1%), and free access was perceived as an important factor by 67 participants (69.8%). Regarding perceived clinical impact, most respondents agreed that digital medical resources improved clinical efficiency, 59 (61.5%), and frequent improvement in patient outcomes was reported by 80 (83.3%). When confidence in managing clinical duties without digital medical resources was assessed, “somewhat confident” responses were most common, 52 (54.2%). The outlook on future digital integration was generally positive, with cautious optimism reported by 46 (47.9%). Pragmatic utilization was the most common overall attitude toward digital resource use, 47 (49.0%). Regarding the doctor-patient relationship, 49 participants (51.0%) reported that digital medical resource use enhances the overall doctor-patient relationship, while 43 (44.8%) were neutral and 4 (4.2%) disagreed. The most commonly reported barrier to digital medical resource utilization was workflow disruption and time constraints, 50 (52.0%).

**Table 2:** Descriptive characteristics and digital medical resource utilization patterns among participants (N = 96). *CME: Continuing Medical Education; AAFP: American Academy of Family Physicians; BMJ: British Medical Journal; MDCalc: Medical Calculator.

Characteristic	Category	n (%)
Frequency of digital medical resource usage during clinic	Daily	43 (44.8%)
	Every other day	22 (22.9%)
	Weekly	21 (21.9%)
	Monthly	8 (8.3%)
	Rarely	2 (2.1%)
Reasons for preferring specific digital medical resources	Illustrative materials (algorithms, flowcharts, etc.)	36 (37.5%)
	Efficient search algorithm	23 (24.0%)
	Simplified language	23 (24.0%)
	Specialty-tailored content	12 (12.5%)
	Evidence-based updates	2 (2.1%)
Digital resource usage duration per session	1-3 minutes	17 (17.7%)
	4-6 minutes	55 (57.3%)
	7-10 minutes	18 (18.8%)
	More than 10 minutes	6 (6.2%)
Digital medical resources used by participants	UpToDate	36 (37.5%)
	AMBOSS	19 (19.8%)
	DynaMed	12 (12.5%)
	BMJ Best Practice	10 (10.4%)
	AAFP	7 (7.3%)
	Medscape	6 (6.2%)
	MDCalc	6 (6.2%)
Primary reasons for using digital medical resources	Access to current medical information	55 (57.3%)
	Enhancing clinical decision-making	37 (38.5%)
	Improving time efficiency	4 (4.2%)
Primary motivators for using digital medical resources	Quality improvement and patient safety	48 (50.0%)
	Aiding recall and reassurance	34 (35.4%)
	Ease of access and availability	14 (14.6%)
Influence of CME* credits on digital medical resource utilization	Agree	51 (53.1%)
	Neutral	39 (40.6%)
	Disagree	6 (6.3%)
Impact of free access	Agree	67 (69.8%)
	Neutral	25 (26.0%)
	Disagree	4 (4.2%)
Perceived clinical efficiency	Agree	59 (61.5%)
	Neutral	31 (32.3%)
	Disagree	6 (6.3%)
Perceived improvement in patient outcomes	Frequent	80 (83.3%)
	Occasional	12 (12.5%)
	No benefit	4 (4.2%)
Confidence in managing clinical duties without digital medical resources	Somewhat unconfident	2 (2.1%)
	Neutral	23 (24.0%)
	Somewhat confident	52 (54.2%)
	Very confident	19 (19.8%)
Outlook on digital integration	Cautious optimism	46 (47.9%)
	Enthusiastic embrace	33 (34.4%)
	Neutral	15 (15.6%)
	Skepticism	2 (2.1%)
Overall attitude toward usage	Pragmatic utilization	47 (49.0%)
	Strong advocacy	28 (29.2%)
	Neutral	15 (15.6%)
	Skepticism/caution	6 (6.3%)
Perceived effect on the doctor-patient relationship	Enhances relationship	49 (51.0%)
	Neutral	43 (44.8%)
	Disagree	4 (4.2%)
Barriers to digital medical resource utilization	Technical difficulties	2 (2.1%)
	User interface and experience issues	11 (11.5%)
	Financial limitations	33 (34.4%)
	Workflow disruption and time constraints	50 (52.0%)

Chi-square analysis was performed to explore associations between age and various aspects of digital resource utilization. Statistically significant associations were observed between age and frequency of resource use (χ² = 36.798, df = 12, p < 0.001), type of digital resource used (χ² = 32.871, df = 18, p = 0.0173), motivation related to free access (χ² = 12.757, df = 6, p = 0.0471), and perceived impact on patient trust (χ² = 22.208, df = 6, p = 0.0011). No significant associations were found for perceptions of integration (χ² = 11.807, df = 9, p = 0.2244) or overall attitudes toward use (χ² = 12.554, df = 9, p = 0.1839), as shown in Table 3.

**Table 3:** Association between age and digital medical resource utilization (N = 96). χ²: Chi-square statistic; df: Degrees of freedom; p-value: Probability value; AAFP: American Academy of Family Physicians; BMJ: British Medical Journal; MDCalc: Medical Calculator.

Variable tested	Response category	25-34 years (n = 61)	35-45 years (n = 26)	46-55 years (n = 7)	>56 years (n = 2)	χ²	df	p-value
Frequency of resource use	Daily	34 (55.7%)	8 (30.8%)	1 (14.3%)	0 (0.0%)	36.8	12	0.0002
	Every other day	10 (16.4%)	10 (38.5%)	2 (28.6%)	0 (0.0%)			
	Weekly	13 (21.3%)	6 (23.1%)	2 (28.6%)	0 (0.0%)			
	Monthly	2 (3.3%)	2 (7.7%)	2 (28.6%)	2 (100.0%)			
	Rarely	2 (3.3%)	0 (0.0%)	0 (0.0%)	0 (0.0%)			
Type of digital resource used	UpToDate	22 (36.1%)	10 (38.5%)	2 (28.6%)	2 (100.0%)	32.87	18	0.0173
	AMBOSS	17 (27.9%)	0 (0.0%)	2 (28.6%)	0 (0.0%)			
	DynaMed	6 (9.8%)	3 (11.5%)	3 (42.9%)	0 (0.0%)			
	BMJ Best Practice	6 (9.8%)	4 (15.4%)	0 (0.0%)	0 (0.0%)			
	AAFP	2 (3.3%)	5 (19.2%)	0 (0.0%)	0 (0.0%)			
	MDCalc	6 (9.8%)	0 (0.0%)	0 (0.0%)	0 (0.0%)			
	Medscape	2 (3.3%)	4 (15.4%)	0 (0.0%)	0 (0.0%)			
Motivation by free access	Agree	38 (62.3%)	24 (92.3%)	3 (42.9%)	2 (100.0%)	12.76	6	0.0471
	Neutral	19 (31.1%)	2 (7.7%)	4 (57.1%)	0 (0.0%)			
	Disagree	4 (6.6%)	0 (0.0%)	0 (0.0%)	0 (0.0%)			
Perceived effect on patient trust	Agree	21 (34.4%)	4 (15.4%)	2 (28.6%)	0 (0.0%)	22.21	6	0.0011
	Neutral	36 (59.0%)	18 (69.2%)	5 (71.4%)	0 (0.0%)			
	Disagree	4 (6.6%)	4 (15.4%)	0 (0.0%)	2 (100.0%)			
Perception of integration	Cautious optimism	33 (54.1%)	7 (26.9%)	4 (57.1%)	2 (100.0%)	11.81	9	0.2244
	Enthusiastic embrace	16 (26.2%)	14 (53.8%)	3 (42.9%)	0 (0.0%)			
	Neutral	10 (16.4%)	5 (19.2%)	0 (0.0%)	0 (0.0%)			
	Skepticism	2 (3.3%)	0 (0.0%)	0 (0.0%)	0 (0.0%)			
Overall attitudes toward use	Pragmatic utilization	33 (54.1%)	10 (38.5%)	4 (57.1%)	0 (0.0%)	12.55	9	0.1839
	Strong advocacy	12 (19.7%)	12 (46.2%)	2 (28.6%)	2 (100.0%)			
	Neutral	12 (19.7%)	2 (7.7%)	1 (14.3%)	0 (0.0%)			
	Skepticism and caution	4 (6.6%)	2 (7.7%)	0 (0.0%)	0 (0.0%)			

Further chi-square analysis assessed the relationship between gender and digital resource utilization. Significant associations were found for frequency of resource use (χ² = 11.103, df = 4, p = 0.0254), type of digital resource used (χ² = 25.818, df = 6, p < 0.001), primary reasons for using digital resources (χ² = 13.247, df = 2, p = 0.0013), reasons for selecting a specific resource (χ² = 16.083, df = 4, p = 0.0029), perceptions of healthcare efficiency (χ² = 11.457, df = 2, p = 0.0033), confidence without digital access (χ² = 15.248, df = 3, p = 0.0016), overall attitudes toward use (χ² = 23.522, df = 3, p < 0.001), perceived barriers or challenges (χ² = 9.589, df = 3, p = 0.0224), and perceived effect on patient trust (χ² = 11.167, df = 2, p = 0.0038). No significant associations were found for duration of resource use, CME credit accumulation, motivation related to free access, observed clinical improvements, or perceptions of integration, as shown in Table 4.

**Table 4:** Association between gender and digital medical resource utilization (N = 96). df: Degrees of freedom; p-value: Probability value; CME: Continuing Medical Education; AAFP: American Academy of Family Physicians; BMJ: British Medical Journal; MDCalc: Medical Calculator.

Variable tested	Response category	Male, n = 55 (57.3%)	Female, n = 41 (42.7%)	χ²	df	p-value
Frequency of resource use	Daily	25 (45.5%)	18 (43.9%)	11.103	4	0.0254
	Every other day	12 (21.8%)	10 (24.4%)			
	Weekly	12 (21.8%)	9 (22.0%)			
	Monthly	4 (7.3%)	4 (9.8%)			
	Rarely	2 (3.6%)	0 (0.0%)			
Type of digital resource used	UpToDate	24 (43.6%)	12 (29.3%)	25.818	6	0.0002
	AMBOSS	10 (18.2%)	9 (22.0%)			
	DynaMed	9 (16.4%)	3 (7.3%)			
	BMJ Best Practice	6 (10.9%)	4 (9.8%)			
	AAFP (American Academy of Family Physicians)	2 (3.6%)	5 (12.2%)			
	Medscape	2 (3.6%)	4 (9.8%)			
	MDCalc	2 (3.6%)	4 (9.8%)			
Primary reasons for using digital resources	Access to up-to-date medical information	28 (50.9%)	27 (65.9%)	13.247	2	0.0013
	Improved clinical decision-making	27 (49.1%)	10 (24.4%)			
	Time efficiency/recalling medical information	0 (0.0%)	4 (9.8%)			
Reasons for selecting a specific resource	Availability of illustrative materials, e.g., flowcharts, algorithms, and images	18 (32.7%)	18 (43.9%)	16.083	4	0.0029
	Easy search algorithm	13 (23.6%)	10 (24.4%)			
	Simple language	16 (29.1%)	7 (17.1%)			
	Stratified presentation of medical information based on physician specialty and patient care	6 (10.9%)	6 (14.6%)			
	Up-to-date and evidence-based medical practice	2 (3.6%)	0 (0.0%)			
Perceptions of healthcare efficiency	Agree	29 (52.7%)	30 (73.2%)	11.457	2	0.0033
	Neutral	22 (40.0%)	9 (22.0%)			
	Disagree	4 (7.3%)	2 (4.9%)			
Confidence without digital access	Somewhat unconfident	0 (0.0%)	2 (4.9%)	15.248	3	0.0016
	Neutral	16 (29.1%)	7 (17.1%)			
	Somewhat confident	27 (49.1%)	25 (61.0%)			
	Very confident	12 (21.8%)	7 (17.1%)			
Overall attitudes toward use	Pragmatic utilization	29 (52.7%)	18 (43.9%)	23.522	3	<0.001
	Strong advocacy	14 (25.5%)	14 (34.1%)			
	Neutral	8 (14.5%)	7 (17.1%)			
	Skepticism and caution	4 (7.3%)	2 (4.9%)			
Perceived barriers/challenges	Workflow disruption and time constraints	25 (45.5%)	25 (61.0%)	9.589	3	0.0224
	Cost and financial constraints	24 (43.6%)	9 (22.0%)			
	User experience and interface design	6 (10.9%)	5 (12.2%)			
	Technical issues	0 (0.0%)	2 (4.9%)			
Perceived effect on patient trust	Agree	16 (29.1%)	11 (26.8%)	11.167	2	0.0038
	Neutral	33 (60.0%)	26 (63.4%)			
	Disagree	6 (10.9%)	4 (9.8%)			
Duration of resource use	1-3 minutes	10 (18.2%)	7 (17.1%)	6.111	3	0.1063
	4-6 minutes	31 (56.4%)	24 (58.5%)			
	7-10 minutes	10 (18.2%)	8 (19.5%)			
	More than 10 minutes	4 (7.3%)	2 (4.9%)			
CME credit accumulation	Agree	29 (52.7%)	22 (53.7%)	0.665	2	0.717
	Neutral	20 (36.4%)	19 (46.3%)			
	Disagree	6 (10.9%)	0 (0.0%)			
Motivation by free access	Agree	35 (63.6%)	32 (78.0%)	5.581	2	0.0614
	Neutral	18 (32.7%)	7 (17.1%)			
	Disagree	2 (3.6%)	2 (4.9%)			
Observed clinical improvements	Yes, always	21 (38.2%)	16 (39.0%)	1.253	3	0.7404
	Yes, frequently	20 (36.4%)	23 (56.1%)			
	Yes, occasionally	10 (18.2%)	2 (4.9%)			
	Not yet	4 (7.3%)	0 (0.0%)			
Perception of integration	Cautious optimism	25 (45.5%)	21 (51.2%)	6.183	3	0.103
	Enthusiastic embrace	20 (36.4%)	13 (31.7%)			
	Neutral	10 (18.2%)	5 (12.2%)			
	Skepticism	0 (0.0%)	2 (4.9%)			

Lastly, chi-square analysis of years of clinical experience revealed significant associations with frequency of resource use (χ² = 49.368, df = 16, p < 0.001), duration of resource use (χ² = 39.885, df = 12, p = 0.0001), and type of digital resource used (χ² = 51.243, df = 24, p = 0.0010). No significant association was found for confidence without access to digital resources (χ² = 19.439, df = 12, p = 0.0785), as shown in Table 5.

**Table 5:** Association between clinical experience and digital medical resource utilization (N = 96). df: Degrees of freedom; p-value: Probability value; AAFP: American Academy of Family Physicians; BMJ: British Medical Journal; MDCalc: Medical Calculator.

Variable tested	Response category	<1 year, n = 12 (12.5%)	1-5 years, n = 50 (52.0%)	6-15 years, n = 23 (24.0%)	16-25 years, n = 9 (9.4%)	>25 years, n = 2 (2.1%)	χ²	df	p-value
Frequency of resource use	Daily	8 (66.7%)	29 (58.0%)	5 (21.7%)	1 (11.1%)	0 (0.0%)	49.368	16	<0.001
	Every other day	0 (0.0%)	12 (24.0%)	8 (34.8%)	2 (22.2%)	0 (0.0%)			
	Weekly	4 (33.3%)	7 (14.0%)	6 (26.1%)	4 (44.4%)	0 (0.0%)			
	Monthly	0 (0.0%)	2 (4.0%)	2 (8.7%)	2 (22.2%)	2 (100.0%)			
	Rarely	0 (0.0%)	0 (0.0%)	2 (8.7%)	0 (0.0%)	0 (0.0%)			
Duration of resource use	1-3 minutes	6 (50.0%)	6 (12.0%)	5 (21.7%)	0 (0.0%)	0 (0.0%)	39.885	12	0.0001
	4-6 minutes	6 (50.0%)	36 (72.0%)	10 (43.5%)	3 (33.3%)	0 (0.0%)			
	7-10 minutes	0 (0.0%)	4 (8.0%)	8 (34.8%)	4 (44.4%)	2 (100.0%)			
	More than 10 minutes	0 (0.0%)	4 (8.0%)	0 (0.0%)	2 (22.2%)	0 (0.0%)			
Type of digital resource used	UpToDate	2 (16.7%)	18 (36.0%)	10 (43.5%)	4 (44.4%)	2 (100.0%)	51.243	24	0.001
	AMBOSS	6 (50.0%)	11 (22.0%)	0 (0.0%)	2 (22.2%)	0 (0.0%)			
	DynaMed	2 (16.7%)	7 (14.0%)	0 (0.0%)	3 (33.3%)	0 (0.0%)			
	BMJ Best Practice	2 (16.7%)	4 (8.0%)	4 (17.4%)	0 (0.0%)	0 (0.0%)			
	AAFP	0 (0.0%)	4 (8.0%)	3 (13.0%)	0 (0.0%)	0 (0.0%)			
	Medscape	0 (0.0%)	0 (0.0%)	6 (26.1%)	0 (0.0%)	0 (0.0%)			
	MDCalc	0 (0.0%)	6 (12.0%)	0 (0.0%)	0 (0.0%)	0 (0.0%)			
Confidence without access	Somewhat unconfident	0 (0.0%)	2 (4.0%)	0 (0.0%)	0 (0.0%)	0 (0.0%)	19.439	12	0.0785
	Neutral	4 (33.3%)	11 (22.0%)	8 (34.8%)	0 (0.0%)	0 (0.0%)			
	Somewhat confident	8 (66.7%)	29 (58.0%)	9 (39.1%)	6 (66.7%)	0 (0.0%)			
	Very confident	0 (0.0%)	8 (16.0%)	6 (26.1%)	3 (33.3%)	2 (100.0%)			

## Discussion

This study represents one of the first comprehensive investigations into the utilization patterns, motivations, and perceived barriers related to digital medical resources among board-certified family medicine physicians practicing within the Riyadh Health Clusters. The findings provide valuable insight into how digital health technologies are being integrated into routine clinical workflows by family physicians in Saudi Arabia and offer relevant comparisons with existing literature globally.

The present study revealed that a substantial proportion of family physicians frequently utilize digital medical resources during clinic hours, with 44.8% of respondents reporting daily use and 89.6% using these tools at least weekly, including daily, every-other-day, or weekly use. This high prevalence aligns with findings from international studies, which have demonstrated widespread adoption of point-of-care digital resources among primary care physicians due to their ability to enhance diagnostic accuracy, reduce clinical uncertainty, and support evidence-based decision-making [10,11]. The frequent reliance on digital resources by Saudi family physicians may reflect both increasing comfort with digital tools and alignment with the Kingdom’s broader healthcare digitization efforts under Vision 2030 [12].

Consistent with previous literature, UpToDate was identified as the most commonly utilized digital resource, 37.5%, followed by AMBOSS, 19.79%; DynaMed, 12.5%; BMJ Best Practice, 10.42%; AAFP, 7.29%; Medscape, 6.25%; and MDCalc, 6.25%. Similar trends have been reported among physicians in Western healthcare systems, where UpToDate is often favored for its comprehensiveness, frequent updates, and structured clinical algorithms [13,14]. Notably, the present study also highlights the growing role of alternative platforms such as AMBOSS, which may be increasingly attractive due to their user interface, examination-style content, and visual aids, features particularly valued by younger physicians and those with recent training exposure.

The most frequently cited motivation for using digital resources was access to up-to-date clinical information, 57.29%, followed by enhancement of clinical decision-making, 38.54%, and improvement in time efficiency, 4.17%. This is consistent with the widely recognized role of digital tools in closing evidence-to-practice gaps and providing physicians with rapid access to current guidelines [15]. Interestingly, the study also found that factors such as visual presentation of information, 37.5%; efficient search algorithms, 24.0%; simplified language, 24.0%; specialty-tailored content, 12.5%; and evidence-based updates, 2.1%, played a significant role in influencing physicians’ selection of specific digital platforms. These findings echo earlier work emphasizing the importance of usability, accessibility, and cognitive load reduction in the design of effective clinical decision-support systems [16].

A particularly noteworthy finding of this study was the significant association between demographic factors, including age, job position, and years of clinical experience, and digital resource usage patterns. Younger physicians and those with fewer years of clinical experience demonstrated significantly higher utilization rates and stronger reliance on digital resources for clinical decision-making. These findings align with previous studies suggesting that digital resource dependency may be higher among early-career clinicians, who may be more digitally native, less reliant on experiential knowledge, and more likely to actively seek real-time evidence [17,18]. In contrast, more experienced consultants appeared to demonstrate greater clinical confidence and a somewhat lower frequency of resource use, although not uniformly.

The study also highlights the relevance of institutional and systemic factors in shaping utilization patterns. A total of 53.1% of participants acknowledged that SCFHS-accredited CME credits influenced their utilization of digital resources, and the availability of free institutional access emerged as a significant driver for 69.8% of respondents. These observations underscore the importance of national licensing policies and institutional subscriptions in promoting the adoption of evidence-based digital tools.

Importantly, 61.5% of participants perceived digital resources as improving healthcare efficiency, while 83.3% reported frequent or consistent improvements in patient care outcomes attributed to digital resource utilization. These findings support the accumulating global evidence linking clinical decision-support systems with improved guideline adherence and patient safety [19,20]. Nevertheless, barriers remain: workflow disruption and time constraints were cited by 52.08% of participants, while financial limitations were identified by 34.38%. These results echo longstanding concerns in the literature regarding integration challenges [21]. Although most respondents were neutral or did not report that digital resource use undermined patient trust, a minority expressed concerns, highlighting a potential area for further investigation into patient-centered perspectives on technology use during consultations.

This study has several strengths, including its clear cross-sectional design, defined study timeframe, focused population of board-certified family medicine physicians, structured site-visit data collection approach across multiple Riyadh Health Clusters, and comprehensive assessment of several aspects of digital clinical information and decision-support resource utilization. However, several limitations must be acknowledged. First, the cross-sectional design provides a snapshot of digital resource adoption and utilization between January and May 2025 and does not allow assessment of adoption trajectories, temporal changes, or causal relationships. Second, the findings may have limited generalizability because the study was conducted only among board-certified family medicine physicians working in secondary and tertiary healthcare facilities within Riyadh Health Clusters 1, 2, and 3, in Riyadh, Saudi Arabia. Therefore, the findings may not fully represent physicians working in other regions, primary healthcare centers, private-sector facilities, or other healthcare systems. Third, although the achieved sample size was statistically adequate for the target population, the structured time-based non-random sampling approach may introduce selection bias, as participation depended on physician availability during site visits. Fourth, this study assessed the use of standalone digital clinical information and decision-support resources, such as UpToDate, AMBOSS, DynaMed, BMJ Best Practice, AAFP, Medscape, and MDCalc; however, it did not evaluate clinical decision-support systems integrated into electronic medical record platforms. Finally, although the broader role of artificial intelligence in medicine was discussed in the literature, this study did not specifically assess the use of artificial intelligence-based resources or platforms, such as OpenEvidence, VisualDx, GI Genius, PETAL, or similar tools.

Future research should consider longitudinal designs or repeated cross-sectional studies to explore how digital resource usage evolves over time as clinicians progress through different stages of their careers. Future studies should also evaluate the adoption of electronic medical record-integrated clinical decision-support systems and artificial intelligence-based tools, as these platforms may increasingly influence digital resource utilization in clinical practice. Additionally, qualitative studies may provide richer insights into physicians’ lived experiences, perceived barriers, and patient perspectives regarding digital resource use at the point of care. Expanding similar research to other specialties and geographic regions across the Kingdom would also enhance generalizability and inform broader health system planning.

## Conclusions

This study provides one of the first comprehensive evaluations of digital medical resource utilization among board-certified family medicine physicians in Saudi Arabia, specifically within the Riyadh Health Clusters. The findings demonstrate that digital resources are widely integrated into clinical practice, with younger and less experienced physicians showing a higher frequency of use and greater reliance on these tools during clinical decision-making. UpToDate remains the most frequently utilized resource, although alternative platforms are increasingly being adopted, influenced by factors such as usability, visual aids, and ease of access.

The integration of digital resources was perceived by most participants to enhance clinical efficiency, improve patient outcomes, and support evidence-based practice. However, persistent barriers, including time constraints, workflow disruptions, and financial limitations, highlight the need for continuous efforts to optimize resource integration within clinical workflows.

These results emphasize the critical role of institutional policies, professional development programs, and national initiatives such as SCFHS accreditation in promoting the adoption of high-quality digital tools. Future research should explore longitudinal usage trends, investigate specialty-specific patterns, and incorporate patient-centered perspectives to further guide the effective implementation of digital resources in primary care settings across Saudi Arabia.

## Appendices

Appendix 1

Questionnaire Tool

Age

☐ 25-34 years ☐ 35-45 years ☐ 46-55 years ☐ 56 years and above

Gender

☐ Male ☐ Female

Years of clinical experience

☐ Less than 1 year ☐ 1-5 years ☐ 6-15 years ☐ 16-25 years ☐ More than 25 years

Job position based on SCFHS classification

☐ Consultant ☐ Senior registrar/specialist

Do you use digital medical resources during your clinical practice? *

☐ Yes ☐ No → If selected, please do not proceed with the rest of the questionnaire. Thank you for your time.

How frequently do you use digital medical resources during clinic hours?

☐ Daily ☐ Every other day ☐ Weekly ☐ Monthly ☐ Rarely

How much time do you usually spend per use?

☐ 1-3 minutes ☐ 4-6 minutes ☐ 7-10 minutes ☐ More than 10 minutes

Which digital resources do you use most often during clinic hours?

☐ UpToDate ☐ AMBOSS ☐ DynaMed ☐ BMJ Best Practice ☐ AAFP ☐ Daleel FM ☐ MDCalc ☐ Medscape ☐ Other: ____________

What are the main reasons for using digital resources during clinic hours?

☐ Access to up-to-date medical information ☐ Improved clinical decision-making ☐ Time efficiency ☐ Other: ____________

What are the main motivating factors that encourage you to adopt digital resources in clinical practice?

☐ Quality improvement and patient safety ☐ Recall and reassurance ☐ Ease of access and availability ☐ Other: ____________

What are the main reasons for using a specific digital resource during clinic hours?

☐ Availability of illustrations ☐ Simple language ☐ Easy search algorithm ☐ Stratified medical information ☐ CME accreditation ☐ Other: ____________

Does the accumulation of CME hours, accredited by SCFHS, play an important role in your use of digital medical resources?

☐ Agree ☐ Neutral ☐ Disagree

Does free access to digital medical resources motivate you to use them in your clinic?

☐ Agree ☐ Neutral ☐ Disagree

Has the use of digital resources improved healthcare delivery efficiency and effectiveness?

☐ Agree ☐ Neutral ☐ Disagree

Have you observed specific improvements in patient care or clinical outcomes as a result of using digital resources?

☐ Yes, always ☐ Yes, frequently ☐ Yes, occasionally ☐ Not yet

How confident are you in working in a clinic without access to digital medical resources?

☐ Very confident ☐ Somewhat confident ☐ Neutral ☐ Somewhat unconfident ☐ Very unconfident

How do you perceive the integration of digital resources into clinical practice?

☐ Enthusiastic embrace ☐ Cautious optimism ☐ Neutral ☐ Skepticism ☐ Limited integration

What is your overall attitude toward using digital resources during clinic hours?

☐ Strong advocacy ☐ Pragmatic utilization ☐ Neutral ☐ Skepticism and caution

Do you believe digital resource use enhances the doctor-patient relationship?

☐ Agree ☐ Neutral ☐ Disagree

What are the main barriers or challenges you face in using digital resources during clinic hours?

☐ Cost and financial constraints ☐ Workflow disruption ☐ User interface issues ☐ Technical difficulties ☐ Other: ____________

Does using digital resources in the presence of patients undermine their trust in the doctor?

☐ Agree ☐ Neutral ☐ Disagree
